# Element-level features in conjoint episodes in dual-tasking

**DOI:** 10.1007/s00426-022-01713-8

**Published:** 2022-08-10

**Authors:** Lasse Pelzer, Christoph Naefgen, Robert Gaschler, Hilde Haider

**Affiliations:** 1 Department of Psychology, University of Cologne, Richard-Strauss-Str. 2, 50931 Cologne, Germany; 2 Department of Psychology, FernUniversität in Hagen, Hagen, Germany

## Abstract

The usual way of thinking about dual-tasking is that the participants represent the two tasks separately. However, several findings suggest that the participants rather seem to integrate the elements of both tasks into a conjoint episode. In three experiments, we aimed at further testing this task integration account in dual-tasking. To this end, we investigated how the processing of the previous Trial n-1 shapes the processing of the current Trial n. We observed performance benefits when the stimulus–response mappings of both tasks repeat in consecutive trials (full repetition: FR) as compared to when only one such mapping repeats (partial repetition: PR). In particular, our experiments focused on the question which elements of the two tasks in dual-tasking might be bound together. For this purpose, in Experiments 1 and 2, all participants performed a dual-task consisting of a visual–manual search task (VST) and an auditory–manual discrimination task (ADT). In the VST the stimulus–response mappings were variable, so that none of the stimuli of this task systematically predicted a certain response. In Experiment 1, the stimuli and responses of the VST were either both repeated or both changed in consecutive trials. In Experiment 2, we removed the stimulus repetitions in the VST and only the responses repeated across trials. In Experiment 3, we changed the ADT into a visual–auditory matching task (VAMT) with variable stimulus–response mappings, so that in both tasks only the responses repeated across trials. In Experiments 1 and 2, we observed better performance for FR than for PR, while this difference disappeared in Experiment 3. Together, the results suggest that the stimulus of one task is sufficient to retrieve the entire episode from the previous trial.

## Introduction

In our daily life, we are used to do more than one thing at a time. For instance, it is quite common nowadays to phone somebody using hands-free devices while biking or driving a car. Even simple things like cooking, doing laundry, or shopping often require us to do multiple tasks at the same time. Thus, the ability to perform two tasks simultaneously seems to gain more and more importance in our daily life. Unfortunately, dual-tasking usually comes with a performance cost (for a current review see Koch et al., 2018).

In the cognitive dual-tasking literature, there is still some debate over the source of these performance costs. There are two currently dominant categories of models that aim to explain these dual-task costs: Bottleneck models, on the one hand, and capacity sharing models, on the other hand. From the perspective of bottleneck models, the central processing stage of response selection is assumed to be limited such that two response selection processes cannot run in parallel. Some authors assume that this bottleneck arises from structural limitations (Pashler, 1994), while other authors assume that performing the two tasks in a serial manner represents a strategic adaptation to avoid crosstalk between the tasks (Logan & Gordon, 2001; Meyer & Kieras, 1997). In contrast to structural bottleneck models, in capacity sharing models it is supposed that the response selection can run in parallel, but a limited pool of central resources has to be shared between both tasks, causing again a processing limitation (Kahneman, 1973; Navon & Miller, 2002; Telford, 1931; Tombu & Jolicoeur, 2003; Welford, 1952). Despite these differences, both classes of models centrally focus on processing limitations on the response selection stage as the source of dual-task interference.

In the meantime, one additional line of research in dual-tasking focuses on questions concerning the representation of the two tasks (e.g., Janczyk & Kunde, 2020; Künzell et al., 2018). When thinking about dual-tasking experiments, the participants are usually instructed to perform two tasks within one trial, both requiring a response. These two tasks have to be processed to complete the trial and often feedback is administered only after having responded to both tasks before then the next trial starts (Dreisbach, 2012). Thus, the instruction in dual-tasking experiments might suggest the participants to interpret the two tasks as belonging together. Consequently, it is conceivable that the participants may represent the two tasks as one single task set consisting of two different stimuli and two different responses, at least if the two task stimuli appear in close temporal proximity (Freedberg et al., 2014; Künzell et al., 2018; Schumacher & Hazeltine, 2016). From this perspective of task integration, dual-task interference might then reflect a direct consequence of confusions resulting from the generation of one common task set (Schumacher & Hazeltine, 2016).

Although such a task integration perspective might run counter to the supposedly implicit assumption that a dual-tasking situation consists of two separate task sets (Navon & Miller, 2002; Pashler, 1994), some experimental findings from different research lines already support this view. For instance, Luria and Meiran (2003, 2006) randomly changed the order of task presentation (A → B; B → A) during dual-task training. They obtained substantial costs when the task order changed from one trial to the next. Meanwhile, several findings support this observation (Huestegge et al., 2021; Kübler et al., 2018, 2021; Sigman & Dehaene, 2006; Stelzel et al., 2008; Strobach et al., 2018, 2021).

Slightly different, Hirsch and colleagues (Hirsch et al., 2017, 2018, 2021) changed during dual-task training the specific task set combination. That is, either the particular task set presented as Task 1 (e.g., A → B; C → B) or as Task 2 (e.g., A → B; A → C) were replaced by another one. The authors reported substantial task-pair switching costs. Thus, these findings suggest that not only the order of the two task sets but also the specific task set pairs seem to be represented conjointly in dual-tasking experiments.

In addition to this line of research which could be seen as support for task integration on the level of task sets, two different strands of research exist suggesting that task integration in dual-tasking can also be observed on the level of stimuli and responses (task-element level, hereafter). The first line of support stems from dual-task studies that combine an implicit sequence learning task with a second randomly sequenced task (Schmidtke & Heuer, 1997). In most of these studies, the serial reaction time task (SRTT) is used. In the SRTT, first introduced by Nissen and Bullemer (1987), the participants see four marked locations on the screen which are mapped to respective response keys. In each trial, a stimulus appears at one location on the screen and the participants have to press the assigned response key. Unbeknownst to the participants, the marked screen locations follow a regular and repeating sequence. Even though implicit learning is a generally robust phenomenon (e.g., Reber, 1993), it is often found to be hampered in dual-tasking (Hsiao & Reber, 2001; Rah et al., 2000; Röttger et al., 2019, 2021; Schmidtke & Heuer, 1997; Schumacher & Schwarb, 2009).

In line with the assumption of task integration on the task-element level, Schmidtke and Heuer (1997) proposed that, due to the close temporal proximity of the stimuli of the two tasks, the participants tend to integrate them into a single continuous task stream. They showed that as long as the secondary task consists of randomly sequenced stimuli and responses, integrating the stimuli of both tasks interrupts the learning of the predictable sequence within the SRTT. This suggests that the randomness of the secondary task masks the sequential predictability of the primary task, because stimuli and responses of both tasks are processed and stored in an integrated way.

More recently, Röttger et al. (2019) tested this assumption of Schmidtke and Heuer (1997). They trained the participants with the SRTT and a tone-discrimination task. Importantly, half of the SRTT positions were consistently paired with one specific tone while for the other half, the relation between the SRTT positions and the tones varied randomly. The results revealed that implicit learning was preserved for the fixedly, but hampered for the variably paired SRTT-tone combinations. These findings suggest that sequence learning in dual-tasking might need to take place via an indirect way. The participants need to first learn the compounds of the stimuli and responses of the two tasks within a trial, before they then can account for sequential predictability. This finding nicely fits with the assumption of an integrated task representation in dual-tasking (for further evidence see Röttger et al., 2021).

The second line of support for task integration taking place on the task-element level is based on the logic of feature binding in action control tasks and task switching (cf. Frings et al., 2020; Hommel, 1998; Koch et al., 2018). According to episodic binding accounts, it is assumed that whenever a task is to be conducted, task features of stimulus and response are bound into an event file (Hommel & Colzato, 2009; Hommel & Frings, 2020) or a stimulus–response episode (Frings et al., 2020). If a stimulus is encountered again, the response that was previously associated with this stimulus is automatically re-activated (Hommel, 1998). In case of an identical response in the current trial, the retrieved response facilitates performance. Yet, if the current response differs from the retrieved response, this interference will cause a performance decrement. This frequently observed performance pattern is labeled partial repetition costs (Dreisbach & Haider, 2008; Frings et al., 2020; Geyer et al., 2006; Hillstrom, 2000; Lamy et al., 2011; Zehetleitner et al., 2012).

Pelzer et al. (2021) transferred this logic of partial repetition costs to dual-tasking experiments, because this might provide a rather direct way to investigate the assumption of task integration on the element level. If the participants stored the elements of both tasks as a single episode one should expect to find faster responses when the stimuli and responses of both tasks repeat across consecutive trials (full repetition; FR) than when the stimulus–response mapping of one task repeats while that of the other task changes (partial repetition; PR). In the case of FRs, the entire integrated episode from the preceding trial is re-activated and in turn should facilitate performance. By contrast, in the case of PRs, the previous integrated episode cannot be re-used leading to slower responses.

To investigate this assumption, Pelzer et al. (2021) trained the participants with a visual-manual and an auditory-verbal task. The stimuli of both tasks were concurrently presented and did not follow any sequential regularity. The findings revealed faster responses for FRs than for PRs. This performance difference was robust over the blocks of practice. In a second experiment, the stimuli of the two tasks were consistently paired, such that the participants could learn these contingencies between the stimuli of the two tasks. Here, the performance difference between FRs and PRs disappeared across practice suggesting that the learned contingencies superseded the after-effect of the preceding trial (for similar findings, see Zhao et al., 2020). Thus, the results suggest that task integration on the task-element level is based on rather automatically formed conjoint memory episodes representing the elements of both tasks. We assume a rather automatic storage of these episodes because they are generated for randomly paired as well as for contingently paired task elements. In addition, they seem to contain short-term (i.e., from Trial n–1) and long-term learned pairings as well (Pelzer et al., 2021; Roettger et al., 2021; Zhao et al., 2020).

To summarize, the reported findings from quite different strands of dual-tasking research support the general assumption that within a dual-tasking situation the participants may not handle the two tasks presented in a dual-tasking trial as entirely independent tasks. Rather they seem to represent them as belonging together (Luria & Meiran, 2006) or even generate a conjoint representation for both tasks (Künzell et al., 2018; Schumacher & Hazeltine, 2016). This might take place on the task set level, but also on the task-element level. In the current study, we focus on the task-element level and in particular on the assumption that task integration on the element level relies on the generation of conjoint memory episodes encompassing stimuli and responses of both tasks.

## The present study

The goal of the present study was to further investigate task integration on the element level in dual-tasking. The above-mentioned findings from implicit learning in dual-tasking suggest already that the stimuli of the two tasks need to be integrated into compounds before the participants can account for the sequential predictability. In addition, the results reported by Pelzer et al. (2021) are in line with the assumption that a just processed dual-task trial is stored as a conjoint episode which is re-activated whenever at least one of the two stimuli repeats in the next trial (probably similar to the episodic retrieval account, Frings et al., 2020). However, these findings leave the question *which* elements of the two tasks are exactly associated or bound in such a conjoint episode. The former findings allow for, at least, three possibilities: (a) only the stimuli of the two tasks are associated, which then, due to the stimulus–response mapping within the respective tasks, activate the corresponding responses (cf., Henson et al., 2014); (b) associations between all elements of the two tasks (between the stimuli, the responses as well as between the stimuli and responses) are generated; or (c) only the two responses of the previous trials remain active and are re-activated in the current trial (response repetition effect, cf. Koch et al., 2017; Schuch & Koch, 2004).

**Fig. 1:**
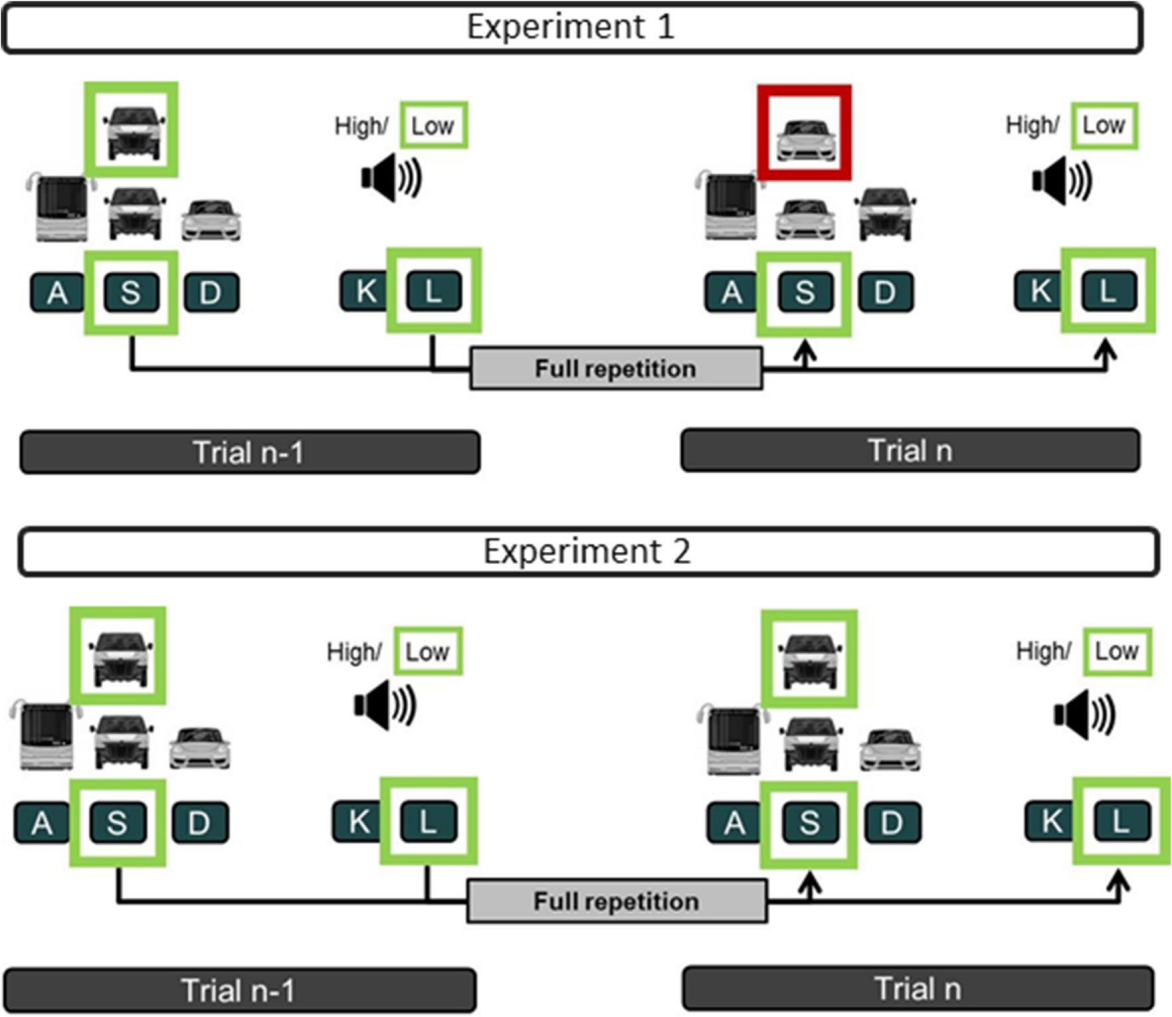
Example of a full repetition in Experiment 1 (upper panel) and Experiment 2 (lower panel)

The former experiments could not answer this question, because, as usually in most experiments in dual-tasking, each stimulus was fixedly mapped to a certain response. Disentangling these stimulus–response mappings (S-R mappings) in one or both tasks should provide one way to investigate this question. To this end, we built on the experimental design of Pelzer et al. (2021) and compared the performance for FRs and PRs from Trial n-1 to Trial n to assess task integration on the element level. Yet, we changed the first task of our experimental design into a visual-manual search task (VST; Fig. 1). The participants saw three different stimuli on the screen. Above them, one of these three stimuli appeared as the target. The participants’ task was to find the target identity among the three stimuli and to press the respective spatially mapped response key. In such a visual search task, the target identity and the location of the three stimuli could vary independently. For instance, the target identity can repeat from the preceding to the current trial, but its response location among the three stimuli changes, or the target identity changes and the response location repeats. Consequently, the target identity is not bound to one specific response and thus will not activate only one single response as it is the case in tasks with fixed S-R mappings (Greenwald, 1970; Koch et al., 2004; Prinz, 1987). With the exception of Experiment 3, the second task of the current experimental design was an auditory–manual tone-discrimination task (ADT; Fig. 1) consisting of a fixed S-R mapping. The stimulus was either a high or a low pitched tone and the participants had to press one certain key for the high and another for the low tone.

This design allowed us to rather systematically investigate which elements of the two tasks are associated and will re-activate information from the previous trial. To this end, we manipulated the VST and the ADT across the three experiments by disentangling the fixed S-R mapping to gradually remove the stimulus information that is repeated across consecutive trials. In Experiments 1 and 2, we manipulated the VST. In Experiment 1, the respective VST target and its response location among the response stimuli always both either repeated or changed between consecutive trials. Thus, here, the VST was rather similar to a task with a fixed S-R mapping. Whenever the target re-appears in the next trial, it leads to the same response as in the previous trial. Therefore, in this experiment, the stimuli of either the VST, the ADT or of both tasks could re-activate the two tasks’ responses of the previous episode. If our considerations concerning episodic retrieval were correct, we should find robust performance benefits for FRs as compared to PRs in Experiment 1. In Experiment 2, we removed the target repetitions in the VST, such that only the response in this task repeated or changed from one trial to the next. If only the stimuli of both tasks were associated which then activate the respective S-R mappings, we should find performance differences between FRs and PRs in Experiment 1, but not in Experiment 2. However, if the stimuli and responses of both tasks are associated, the ADT stimulus should suffice to re-activate the responses of both tasks of the previous episode (Frings et al., 2020). In this case, we should find again benefits for FR in Experiments 1 and 2.

**Fig. 2:**
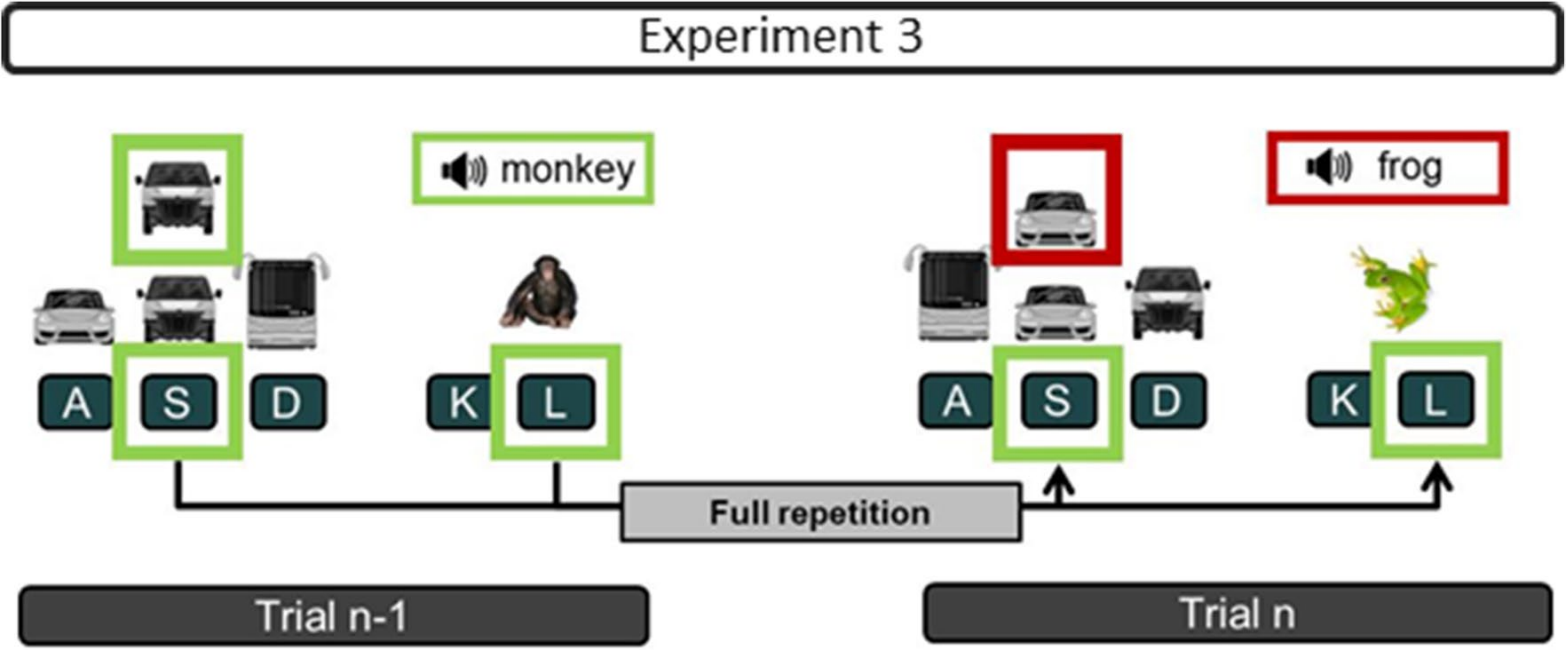
Example of a full repetition in Experiment 3

To test for the third alternative that the performance benefits of the FRs are due to the repetition of the two still active responses, we ran Experiment 3. In this this experiment, we changed the ADT to a visual-auditory matching task (VAMT),^1^ thereby enabling a variable S-R mapping also in this task (see Fig. 2). Thus, here, in both tasks, only the responses repeat from the previous to the current trial. If the performance differences between FRs and PRs result from the repetition of the still active response-pairing from the previous trial, we should find them again in this experiment.

## General method

### Apparatus and stimuli

The experiments were controlled by a custom-written software (implemented in PsychoPy v3.0, Peirce et al., 2019). The two tasks used in the present experiments were modified versions of the dual-task paradigm of Pelzer et al. (2021).

In the VST, three different car images were presented on three horizontally aligned positions on the screen (100 ✕ 100 pixels, separated by gaps of 300 pixels). We always used the three different car images depicted in Fig. 1. The three positions were assigned to spatially mapped response keys (A, S, D on a German QWERTZ keyboard for the left, middle and right position, respectively). A target image (100 ✕ 100 pixels) depicting one of the three car images, appeared 200 pixels above the middle position. Participants were instructed to respond with their left hand (ring, middle and index finger for the three positions from left to right, respectively) as fast and as accurately as possible with the key aligned to the location of the car image representing the target car (Fig. 1).

In Experiments 1 and 2, concurrently with the VST target, either a high (900 Hz) or low pitched tone (300 Hz) sounded in the ADT for 56 ms. The participants had to indicate with their right hand (index and middle finger for the low or high tone, respectively) which tone they heard by pressing the respective key (K for the low and L for the high tone on a German QWERTZ keyboard) (Fig. 1).

In Experiment 3, the VST was identical to that of Experiment 2, but the ADT was changed into the VAMT, so that the stimuli were not contingently bound to a certain response (Fig. 2). In the VAMT, the sound of an animal was concurrently displayed with the image of an animal that appeared on the screen (100 ✕ 100 pixels, 500 pixels to the right of the VST target). The participants had to decide whether the sound of the animal matched the presented image of an animal by either pressing the k- or l-keys on a German QWERTZ keyboard with their right hand (index and middle finger for match or mismatch, respectively). Overall six different animals were used, resulting in 36 possible image-sound pairs, which pseudo-randomly changed in each trial. To avoid any content-dependent overlap between stimuli in consecutive trials (e.g., sound of a dog in Trial n-1 and image of a dog in Trial n), in the current Trial n, we never presented the sound or the image of an animal which was already used in form of a sound or an image in the previous Trial n-1.

### Procedure

All participants were instructed step by step. First, they started with 20 practice trials with only the VST and another 20 practice trials with only the ADT or with only the VAMT in Experiment 3. In the last practice block, they received 20 trials of the dual-task. Immediately after this practice phase, the participants performed 6 dual-task blocks of 108 trials, each. In all experiments, trials and stimulus combinations within trials were presented in a pseudorandomized order. Thus, neither within-task nor across-task regularities were built into the tasks.

In all three experiments, a dual-task trial always began with the presentation of the three different car images at the three positions on the screen. After 200 ms, the VST target (one of the three car images) appeared together with the auditory stimulus. Participants had to first locate the position of the target car in the VST and to indicate which tone they heard in the ADT by pressing the respective keys assigned to the two tasks (Exp. 1 and 2). In Experiment 3, the ADT was changed to the VAMT and the high and low tones were replaced by the sound of an animal presented for 600 ms together with the image of an animal.

In all experiments, participants were encouraged by instruction to first respond to the VST and then to the ADT/VAMT but to give both tasks equal priority, as well as to respond as fast and as accurately as possible. The response-window always closed after both tasks’ responses were entered or after 2200 ms had elapsed. The next trial started after a fixed inter-trial interval of 750 ms.

### Data analysis

Our main goal was to further investigate the task integration account. More specific, we tested which task elements are associated across tasks within a conjoint episode. Therefore, we focused on the performance differences between FRs and PRs with regard to the effect of the previous Trial n-1 on the current Trial n. These trials are the most indicative ones that the participants generated conjoint episodes because in both trials the S-R mapping (or response) of one task repeats while that of the respective other task either also repeats (FR) or changes (PR). For means of completeness, we nevertheless included in our main analyses all four combinations of repetitions and switches of the stimuli and responses in the two tasks.

For each experiment, we conducted separately for the VST as well as for the ADT/VAMT a 2 (Sequence of VST: repetition vs. switch) ✕ 2 (Sequence of ADT/VAMT: repetition vs. switch) repeated measures ANOVA with either reaction times (RTs) or error rates as the dependent variables. In addition, we computed planned interaction contrasts between the respective FR and PR trials separately for the two tasks. That means, we compared the repetitions of the S-R mapping within the VST with the repetitions (FR) or changes (PR_VST_) of the S-R mapping in the ADT/VAMT. In the ADT/VAMT the comparison of trials was analogous, repetitions of the S-R mapping in the ADT/VAMT were compared with repetitions (FR) or changes (PR_ADT/VAMT_) of the S-R mapping in the VST. If the two responses are associated in any way, the respective repetitions from Trial n–1 to Trial n should be faster when the responses of both tasks repeat than when the response in the respective other task switches. By contrast, if only the stimuli are associated and activate the corresponding S-R mappings, we should find a significant effect for these contrasts only in Experiment 1.

To assess the robustness of the results, we computed for all the reported planned interaction contrasts Bayes factors with JASP (JASP Team, 2020), using the Bayesian *t* test framework (Rouder et al., 2009). Here, we conducted Bayesian paired sample t tests separately for the RTs and the error rates testing the one-tailed alternative hypothesis (H_1_), postulating shorter RTs and lower error rates for the FR compared to the respective PR (PR_VST_ or PR_ADT/VAMT_). The default prior option was set to a Cauchy distribution with spread *r* set to 0.707 (Jeffreys, 1961; Rouder et al., 2009).

In all RT analyses, we excluded trials in which an error had occurred in either the VST, the ADT/VAMT, or in both tasks.^2^ Additionally we excluded all trials in which the RTs were shorter than 200 ms or longer than 2000 ms. In addition, the first trial of each block was eliminated since it has no precursor. Lastly, we replaced the data-set of participants who made more than 30% errors in at least one of the six dual-task blocks by that of a new participant.

## Experiment 1

The goal of Experiment 1 was to replicate the Trial n-1 episodic retrieval reported in Pelzer et al. (2021) with the setup to be used in the current study. To this end, the target stimulus and the response in the VST always both repeated or both changed, so that a repetition from Trial n-1 to Trial n, here, refers to an identical target stimulus and its identical response location among the three stimuli. Due to the fixed S-R mapping in the ADT, also in this task, the target stimulus and the response both repeated and changed from Trial n-1 to Trial n. Thus, the design was maximally similar to that of Experiment 1 of Pelzer et al. (2021), except that the first task was a visual search task rather than a visual categorization task. If our considerations concerning episodic retrieval were correct, we should find robust performance benefits for FRs compared to PRs.

## Methods

### Participants

Twenty-three German-speaking participants (14 men, 9 women, mean age years = 26.4, SD = 4.1) were recruited using the crowd sourcing platform Prolific (Palan & Schitter, 2018) and tested online. For the participation in the approximately 30 min long online experiment, the participants received monetary compensation (£6.25). An a priori power analysis revealed that a 2 ✕ 2 repeated measures ANOVA with approximately 20 participants would be sensitive to detect effects of *ηp*^2^ = 0.14 with 90% power (alpha = 0.05).^3^

### Apparatus and stimuli

Apparatus and stimuli were as described in the General Method. In each of the six blocks, the trial distribution was 16.82% FR, 42.06% FS, 24.30% PR_ADT_, and 16.82% PR_VST_. This trial distribution applied to the sequence of stimuli and responses.

## Results and discussion

Overall, 13.90% of the trials were excluded due to an error or as an RT outlier in the VST or the ADT. The data-set of four participants were replaced by a new one due to more than 30 percent incorrect responses in the VST or the ADT.

### Performance in the VST

For the RTs in the VST, the 2 ✕ 2 repeated measures ANOVA revealed significant main effects of Sequence of VST and of Sequence of ADT (see Table 1). Overall, RTs were shorter when the required response in the VST or in the AT repeated than when it switched. The important interaction effect was significant, too (as shown in Table 1). In addition, the planned interaction contrast comparing the FR with the PR_VST_, revealed a significant difference indicating shorter RTs for the FR (915 ms) than for the PR_VST_ (1026 ms), *t*(22) = 8.41, *p* < 0.001, *d* = 1.75 (see Fig. 1). This interaction was further confirmed by strong support for the H_1_ from the Bayes analysis (BF_10_ = 1.077e^6^).

**Table 1:** Results of the 2✕2 repeated measures ANOVAs for the RTs and error rates in the VST in Experiment 1

Effect	RTs			Error rates		
	*F* (1, 22)	*p*	*ηp*^2^	*F* (1, 22)	*p*	*ηp*^2^
Main effect VST	51.51	< 0.001	0.7	11.94	= 0.002	0.35
Main effect ADT	51.4	< 0.001	0.7	5.59	= 0.03	0.2
VST ✕ ADT Interaction	84.52	< 0.001	0.8	1.13	= 0.3	0.05

**Fig. 3:**
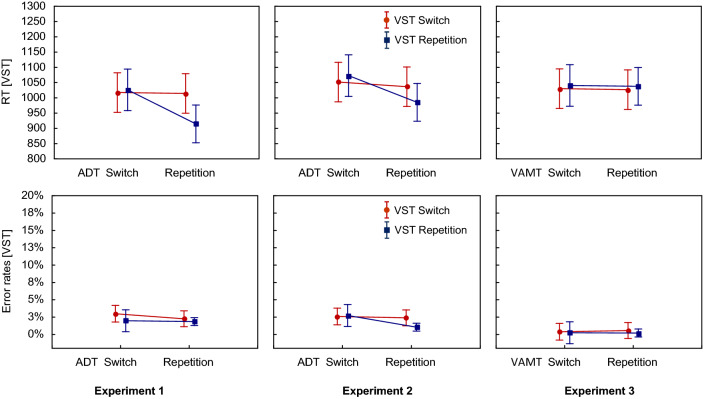
Mean RTs (top row) and error rates (bottom row) for the VST as a function of Sequence of VST (switch vs. repetition) and Sequence of ADT/VAMT (switch vs. repetition) separately for each of the three experiments. Error bars represent the standard errors of means

For the error rates, the analogous ANOVA also revealed significant main effects of Sequence of VST and Sequence of ADT (see Table 1). Overall, participants made fewer errors when the required responses in the VST or in the ADT repeated than when they switched. As can be seen in Table 1, the interaction effect was not significant. The planned interaction contrast also indicated that the error rates did not significantly differ between FR (1.9%) and PR_VST_ (2%), *t*(22) = 0.33, *p* = 0.74, *d* = 0.07 (see Fig. 3). The Bayes factor indicated moderate evidence for the null hypothesis (BF_10_ = 0.29).

### Performance in the ADT

For the RTs in the ADT, the 2 ✕ 2 repeated measures ANOVA revealed significant main effects of Sequence of VST and Sequence of ADT (see Table 2). Again, RTs were shorter when the required responses in the VST or in the ADT repeated than when they switched. Further, the interaction effect was significant (as shown in Table 2). In addition, the planned interaction contrast comparing the FR with the PR_ADT_, revealed significantly shorter RTs for FR (1118 ms) than for PR_ADT_ (1253 ms), *t*(22) = 11.3, *p* < 0.001, *d* = 2.36 (see Fig. 3). In addition, the Bayes factor confirmed the significant difference between FR and PR (BF_10_ = 1.544e^8^).

**Table 2:** Results of the 2✕2 repeated measures ANOVAs for the RTs and error rates in the ADT in Experiment 1

Effect	RTs			Error rates		
	*F* (1, 22)	*p*	*ηp*^2^	*F* (1, 22)	*p*	*ηp*^2^
Main effect VST	80.9	< 0.001	0.79	5.39	= 0.03	0.2
Main effect ADT	98.36	< 0.001	0.82	18.67	< 0.001	0.46
VST ✕ ADT Interaction	117.19	< 0.001	0.84	46.01	< 0.001	0.68

**Fig. 4:**
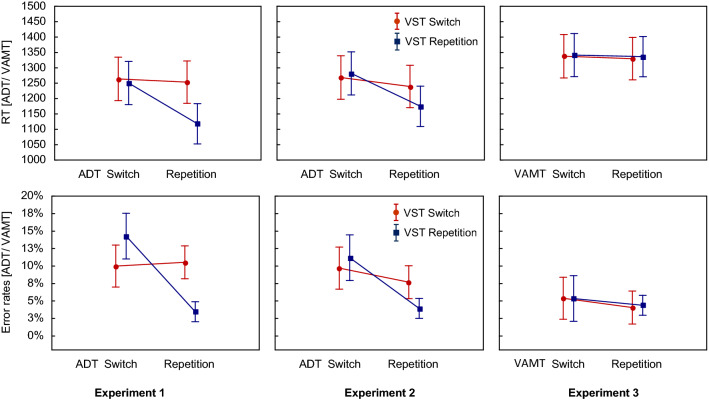
Mean RTs (top row) and error rates (bottom row) for the ADT/VAMT as a function of Sequence of VST (switch vs. repetition) and Sequence of ADT/VAMT (switch vs. repetition) separately for each of the three experiments. Error bars represent the standard errors of means

The 2 ✕ 2 repeated measures ANOVA for the error rates also revealed significant main effects of Sequence of VST and Sequence of ADT (see Table 2). Again, the participants made fewer errors when the required response in the VST or in the ADT repeated than when it switched. As can be seen in Table 2, the interaction effect was significant. The planned interaction contrast revealed a significant difference, indicating lower error rates for FR (3%) than PR_ADT_ (11%), *t*(22) = 6.72, *p* < 0.001, *d* = 1.4 (see Fig. 4). This difference was again confirmed by the Bayes factor (BF_10_ = 37,943).

Together, the results of Experiment 1 are in line with the findings reported in Pelzer et al. (2021), showing robust differences between FRs and PRs in the context of dual-tasking. We observed significant Sequence of VST ✕ Sequence of ADT interactions for the RTs in both tasks and for the error rates in the ADT. In contrast to the results reported in Pelzer et al. (2021), we did not find faster responses for full switches as compared to partial repetitions. Nevertheless, these findings are in line with the assumption of episodic retrieval. However, they are ambiguous regarding the question whether more than only the stimuli of the two tasks are associated. Experiment 2 served to answer this question.

## Experiment 2

Experiment 2 aimed at testing whether repeating only the stimulus in the fixedly mapped ADT would suffice to re-activate the entire episode of Trial n-1 (Frings et al., 2020). For this purpose, we removed the target stimulus repetitions in the VST such that FRs here refer to response repetitions in the VST and the repetition of the S-R mapping in the ADT. The latter task was identical to Experiment 1. Finding performance differences between FRs and PRs once again would suggest that either the stimulus of the ADT is associated with both responses or that these differences result from the repetition of the two responses.

## Methods

### Participants

The recruitment of participants was identical to Experiment 1. We recruited twenty-three German-speaking participants (16 men, 7 women, mean age years = 24.9, SD = 4.3).

### Apparatus and stimuli

Apparatus and stimuli were as described in the General Method. In each of the six blocks, the trial distribution was identical to that of Experiment 1 (16.82% FR, 42.06% FS, 24.30% PR_ADT_, and 16.82% PR_VST_). Importantly, repetitions in the VST referred always to only response repetitions because the target stimuli in the VST never repeated in consecutive trials.

## Results and discussion

Overall, we excluded 12.90% of the trials due to an error or as an RT outlier in the VST or the ADT. The data-set of two participants were replaced by a new one due to more than 30 percent incorrect responses in the VST or the ADT.

### Performance in the VST

For the RTs in the VST, the 2 ✕ 2 repeated measures ANOVA yielded a significant main effect of Sequence of ADT, while the main effect of Sequence of VST just failed the level of significance (see Table 3). RTs were shorter when the required S-R mapping in the ADT repeated than when it switched. Importantly and shown in Table 3, the interaction effect was significant. In addition, the planned interaction contrast comparing the FR with the PR_VST_ indicated also a significant difference. The participants responded faster with FR (985 ms) than with PR_VST_ (1073 ms), *t*(22) = 10.19, *p* < 0.001, *d* = 2.13 (see Fig. 1). This was confirmed by the Bayes analysis providing strong support for the H_1_ (BF_10_ = 2.564e^7^).

**Table 3:** Results of the 2✕2 repeated measures ANOVAs for the RTs and error rates in the VST in Experiment 2

Effect	RTs			Error rates		
	*F* (1, 22)	*p*	*ηp*^2^	*F* (1, 22)	*p*	*ηp*^2^
Main effect VST	3.76	= 0.07	0.15	9.71	= 0.005	0.31
Main effect ADT	123.05	< 0.001	0.85	2.98	= 0.1	0.12
VST ✕ ADT Interaction	50.5	< 0.001	0.7	4.15	= 0.05	0.16

For the error rates, the 2 ✕ 2 repeated measures ANOVA revealed only a significant main effect for Sequence of VST (see Table 3). Here, the participants made fewer errors with response repetitions than with response switches. The interaction effect just missed the level of significance. The interaction contrast showed that the error rates for the PR_VST_ (2.7%) and the FR (1%) did not significantly differ, *t*(22) = 2.01, *p* = 0.06, *d* = 0.42 (see Fig. 1). The Bayes analysis yielded only anecdotal evidence for the H_1_ (BF_10_ = 2.29).

### Performance in the ADT

For the RTs in the ADT, the 2 ✕ 2 repeated measures ANOVA revealed significant main effects of Sequence of VST and Sequence of ADT (see Table 4). Overall, the RTs were shorter when the required responses in the VST or in the ADT repeated than when they switched. Importantly, the interaction effect was significant, as can be seen in Table 4. In addition, the planned interaction contrast comparing the FR with the PR_ADT_ confirmed again the significant difference between the RTs for FR (1175 ms) compared to PR_ADT_ (1239 ms), *t*(22) = 4.88, *p* < 0.001, *d* = 1.02 (see Fig. 2). The Bayes factor (BF_10_ = 748) yielded strong evidence for the H_1_.

**Table 4:** Results of the 2✕2 repeated measures ANOVAs for the RTs and error rates in the ADT in Experiment 2

Effect	RTs			Error rates		
	*F* (1, 22)	*p*	*ηp*^2^	*F* (1, 22)	*p*	*ηp*^2^
Main effect VST	9.65	= 0.005	0.3	4.76	= 0.04	0.18
Main effect ADT	177.52	< 0.001	0.89	33.08	< 0.001	0.6
VST ✕ ADT Interaction	40.05	< 0.001	0.65	28	< 0.001	0.56

For the error rates, the analogous ANOVA revealed significant main effects for both the Sequence of VST and the Sequence of ADT (see Table 4). When the required responses repeated in the VST or in the ADT, the participants made fewer errors than when they switched. As shown in Table 4, the interaction effect was also significant. In addition, the planned interaction contrast confirmed again that the participants made more errors with PR_ADT_ (7.7%) than FR (4%), *t*(22) = 5.06, *p* < 0.001, *d* = 1.06 (see Fig. 2). Again, the Bayes factor indicated strong evidence for this difference (BF_10_ = 1117).

The findings of Experiment 2 were rather similar to those of Experiment 1. In both tasks, the Sequence of VST ✕ Sequence of ADT interactions were again significant—at least for the RTs—as were the planned contrasts between FR and the respective PRs. The finding of performance differences between FRs and PRs even when only the ADT stimulus unambiguously predicts a response is in line with the assumption that the ADT stimulus not only activates the respective ADT response but also the VST response from Trial n–1. However, an alternative explanation is that simply the repetition of the just generated two responses provoked the performance differences between FRs and PRs, in the sense of a simple response repetition effect (Schuch & Koch, 2004). Experiment 3 served to test this alternative explanation.

## Experiment 3

The purpose of Experiment 3 was to test whether the repetition of the responses of the two tasks might have led to the beneficial effect of FR in Experiments 1 and 2. To this end, we changed the ADT into the VAMT, a task with a variable S-R mapping to eliminate also the predictability of the stimulus in this task. Repetitions occurred exclusively at the level of the responses. If the repetition of two responses from Trial n–1 to Trial n in Experiments 1 and 2 had been sufficient to cause the performance differences of FRs compared to PRs, we should obtain them again in this experiment.

## Methods

### Participants

The recruitment was identical to Experiment 1. We recruited twenty-three German-speaking participants (8 men, 15 women, mean age years = 25.4, SD = 5).

### Apparatus and stimuli

Apparatus and stimuli were as described in the General Method. As in the former experiments, the trial distribution was 16.82% FR, 42.06% FS, 24.30% PR of the VAMT and 16.82% PR of the VST. Here, this trial distribution applied only to the sequence of responses, as in both tasks, the targets always switched in consecutive trials.

## Results and discussion

Overall, we excluded 8% of the trials due to an error or a RT outlier in the VST or the VAMT. The data-set of five participants was replaced by a new one because they made more than 30 percent incorrect responses in the VST or the VAMT.

### Performance in the VST

The 2 ✕ 2 repeated measures ANOVA for the RTs revealed a significant main effect of Sequence of VST (see Table 5). However, here, RTs were longer for response repetitions than for response switches. Neither the main effect of Sequence of VAMT nor the interaction were significant. The planned interaction contrast comparing the FR with the PR_VST_, also revealed no significant difference between the FR (1038 ms) and the PR_VST_ (1041 ms), *t*(22) = 0.7, *p* = 0.49, *d* = 0.15 (see Fig. 1).

**Table 5:** Results of the 2✕2 repeated measures ANOVAs for the RTs and error rates in the VST in Experiment 3

Effects	RTs			Error rates		
	*F* (1, 22)	*p*	*ηp*^2^	*F* (1, 22)	*p*	*ηp*^2^
Main effect VST	5.91	= 0.02	0.21	3.16	= 0.09	0.13
Main effect VAMT	1.48	= 0.24	0.06	0.43	= 0.52	0.02
VST ✕ VAMT Interaction	0.01	= 0.92	< 0.001	1.64	= 0.21	0.07

For the error rates, the 2 ✕ 2 repeated measures ANOVA were analogous as there were no significant main effects, neither for Sequence of VST nor for Sequence of VAMT (see Table 5) and no significant interaction. In addition, the planned interaction contrast for the FR (0.2%) versus the PR_VST_ (0.2%) was also insignificant, *t*(22) = 0.27, *p* = 0.79, *d* = 0.06 (see Fig. 1).

Here, we conducted the Bayesian paired sample t tests separately for the RTs and the error rates to test the null hypothesis (H_0_) against the one-tailed alternative hypothesis (H_1_), postulating shorter RTs and lower error rates for FR compared to PR_VST_. The Bayes factors indicated for the H_0_ anecdotal evidence for the RTs (BF_01_ = 2.45) and moderate evidence for the error rates (BF_01_ = 3.68).

### Performance in the VAMT

For the RTs in the VAMT, the 2 ✕ 2 repeated measures ANOVA revealed neither significant main effects nor a significant interaction (see Table 6). The planned interaction contrast comparing the FR with the PR_VAMT_, indicated that the participants did not respond significantly faster in FR trials (1337 ms) than in PR_VAMT_ trials (1330 ms), *t*(22) = 1.65, *p* = 0.11, *d* = 0.34 (see Fig. 2). At least on a numerical level, the RTs for FRs were even longer than for PRs.

**Table 6:** Results of the 2✕2 repeated measures ANOVAs for the RTs and error rates in the VAMT in Experiment 3

Effects	RTs			Error rates		
	*F* (1, 22)	*p*	*ηp*^2^	*F* (1, 22)	*p*	*ηp*^2^
Main effect VST	1.75	= 0.2	0.07	0.14	= 0.71	0.01
Main effect VAMT	3.39	= 0.08	0.13	3.73	= 0.07	0.14
VST ✕ VAMT Interaction	0.26	= 0.61	0.01	0.32	= 0.58	0.01

Also for the error rates, the 2 ✕ 2 repeated measures ANOVA indicated no significant main effects or an interaction (see Table 6). The interaction contrast yielded no significant difference between the FR (4.4%) and the PR_VAMT_ (4.1%), *t*(22) = 0.6, *p* = 0.55, *d* = 0.13 (see Fig. 2).

To also strengthen the results of the planned interaction contrast in the VAMT, we conducted again Bayesian paired sample *t* tests separately for the RTs and the error rates to test the null hypothesis (H_0_), against the one-tailed alternative hypothesis (H_1_), postulating shorter RTs and lower error rates for FR compared to PR_VAMT_. Here, the Bayes factors indicated strong evidence for the null hypothesis for the RTs (BF_01_ = 10.84) and moderate evidence for the error rates (BF_01_ = 6.8).

In sum, the results revealed no significant Sequence of VST × Sequence of VAMT interactions for the RTs or for the error rates in either the VST or the VAMT. Apart from the Bayes factor for the RTs in the VST, this was further supported by moderate to strong Bayes factors in the VST and the VAMT. Thus, the findings point in the direction that a response repetition effect does not suffice to entirely explain the large performance differences between FRs and PRs found in Experiments 1 and 2. Rather, it seems as if it needs at least one reliable retrieval cue to re-activate the conjoint episode from the preceding trial.

## General discussion

The experiments reported here aimed at further testing the task integration account in dual-tasking. Based on the previous findings reported in Pelzer et al. (2021) showing that the participants seem to integrate elements of the two tasks, we focused here on the characteristics of the underlying conjoint task representation. The logic of the experiments was borrowed from episodic retrieval accounts in single tasking (Frings et al., 2020). By implementing a variable S-R mapping in the VST and a fixed S-R mapping in the ADT in the first two experiments and additionally a variable S-R mapping in the VAMT in Experiment 3, we systematically reduced the predictability between the stimulus and the response within the tasks in a dual-task design. This allowed us to test the conditions which might have driven task integration on the element level.

In Experiment 1, the stimulus and the response in the VST either repeated or changed between consecutive trials. Thus, even though the stimuli in the VST were variably mapped to the responses, the same response could be employed whenever the target identity was the same as in the trial before. In Experiment 2, the repetition of the stimulus in the VST was removed such that in case of a FR only the response repeated. In Experiment 3, also in the second task, the VAMT, the stimuli were now variably mapped to the responses. Consequently, a FR consisted of only two repeating responses between consecutive trials.

The results showed reliably faster responses for FRs than for PRs in Experiments 1 and 2 when at least one stimulus of the two tasks was fixedly mapped to a response. If both tasks’ stimuli were variably mapped to the responses, as was the case in Experiment 3, these performance differences were diminished. Thus, the performance differences between FRs and PRs in Experiments 1 and 2 do not seem to reflect a mere response-pair repetition effect from Trial n-1 to Trial n (Schuch & Koch, 2004). Rather, the results seem to be better in line with the assumption that repeating a stimulus which unambiguously predicts its corresponding response additionally activates the response belonging to the other task. This in turn suggests that the conjoint episode does not only contain an association of the two tasks’ stimuli, which then, due to the fixed S-R mapping within the respective tasks, activates the corresponding responses. The current findings suggest that the conjoint episodes contain several associations between the stimuli, the responses and probably also between the stimuli and the responses of the respective other tasks. Alternatively, a more indirect way is also conceivable. A response once triggered activates the response of the respective other task.

Thus overall, the data do not only support our assumption of conjoint episodes representing both tasks of a dual-tasking trial. Furthermore, they also help to better understand what the characteristics of these conjoint episodes are and what task elements are exactly associated across the two tasks in a dual-task.

However, the data need some cautious discussions. First, we observed that the significant interactions in RTs were driven mainly by the benefit of FRs as compared to PRs. The benefit for full switches (FS) was negligible. At first glance, this might be a bit surprising because the current experiments built on those reported in Pelzer et al. (2021), in which the RTs were also faster for FSs than for PRs. The only difference between that study and the current experiments was that the S-R mapping in the VST was changed from a fixed to a variable one. However, this change leads to one probably important consequence. In the experiments reported in Pelzer et al. (2021), each of the three stimuli unambiguously announced one respective response which, according to the Theory of Event Coding (TEC; Hommel & Colzato, 2009), could be represented in three distinct event files that were probably prepared in advance (Hommel & Frings, 2020; Kunde et al., 2004). By contrast, in the current experiments, each of the three different stimuli was combined with the three different response locations leading to 9 different S-R mappings which overlapped in their feature codes. Following the assumptions of TEC, this feature code overlap might have hindered any advance preparation of event files for the VST (Hommel & Müsseler, 2006). This would imply that the participants could not rely on already prepared event files in the VST, but had to generate them whenever the stimulus of the ADT changed (cf. a full switch or a repetition of only the VST stimulus), because, in this case, no unique episode of the previous trial could be re-activated (Gade et al., 2017). This might have caused additional costs (Hommel & Frings, 2020) attenuating the benefits of full switches.

It is important to note that this explanation does not invalidate our main findings concerning the significant FR benefits found in Experiments 1 and 2, but not in Experiment 3. If the participants had represented the two tasks separately, an explanation would be needed why a repetition of the response in one task is only fast when also the response in the respective other task is repeated. Therefore, we believe that the best account for such a performance difference is the assumption of conjoint episodes representing the stimuli and responses of both tasks.

A second point to discuss concerns Experiment 3 where we changed the ADT of Experiments 1 and 2 into a visual–auditory matching task (VAMT) to implement variable S-R mappings in both tasks. We did this, to test whether it needs at least one fixed S-R mapping providing a reliable retrieval cue to re-activate the conjoint episode from the preceding trial or whether the performance difference between FRs and PRs found in Experiments 1 and 2 were due to a mere response repetition effect (Schuch & Koch, 2004). As a side effect, this change likely increased the complexity of the task. Therefore, an alternative explanation for the absence of a performance difference between FR and PR in Experiment 3 could also be that the higher complexity in the VAMT in general prevented any retrieval from memory and thereby attenuated the effect of the still two active responses of the preceding trial. In line with this argument, in the VAMT in Experiment 3, the RTs were longer than in Experiments 1 and 2. However, concurrently the error rates were numerical lower than in Experiments 1 and 2 suggesting that the longer RTs in Experiment 3 might have resulted from a speed–accuracy trade-off rather than from the higher complexity of the VAMT. Besides, this does not directly explain why we also found in the VST no hint for faster responses for FRs than for PRs. We did not change this task from Experiments 1 and 2 to Experiment 3. Furthermore, Moeller and Frings (2019) showed reliable response repetition effects with rather similar types of matching tasks in a prime-probe design comparable to our Trial n–1 to Trial n design. Importantly however, there was one crucial difference between their and our procedures. In their design, the secondary task always appeared after the participants had responded to the first task. This implies that in the probe/Trial n, the response of the first task served as a retrieval cue for the event file containing the secondary tasks’ response of the prime/Trial n-1. In contrast, in our Experiment 3, the tasks were always presented simultaneously, meaning that here the VST response probably could not have activated the response for the VAMT. Because Moeller and Frings (2019) found response repetition effects with a rather similar type of matching task, it is rather unlikely that our findings of Experiment 3 were due to the higher complexity of the VAMT. Nevertheless, since we cannot entirely rule out that such an increase in task complexity could have diminished a response repetition effect, further research is needed to clarify this point.

A third point, important to discuss is that at first sight our current findings are at odds with current results of Kübler et al., (2018, 2021), Kübler, (2021), Strobach et al. (2021) or Hirsch et al. (2021). These experiments differ conceptually from our focus on the task-element level because they investigate on a more abstract task set level the effects of changing task order sets or task-pair sets. For instance, Kübler et al. (2021) or also Strobach et al. (2021) have shown that task order switches led to additional costs in dual-tasking (see also Luria & Meiran, 2006). On the one hand, this fits with our observation that the participants do not handle the two tasks in dual-tasking as isolated task sets. On the other hand, however, at least Kübler (2021) assume, that task set order switch costs result from task order sets actively represented in working memory. These representations do not contain any information about the specific task components. Rather, the specific task sets are assumed to be maintained separately in working memory. In case of such separation, co-occurrence of stimuli and responses of the two tasks in Trial n–1 would not necessarily affect performance in Trial n. One possibility to solve this seemingly contradiction is to assume that randomly switching between different task set orders might have hindered the generation of a conjoint episode during training. However, further research is needed to test this presumption.

Where does these findings leave us? First, together with the additional explanation proposed above they nicely fit the previous results reported in Pelzer et al. (2021). We obtained already robust performance differences between FRs and PRs when we presented the two task stimuli in dual-tasking in random order. When, as we did in our second experiment, the stimuli of Task 1 were fixedly paired with the stimuli of Task 2, these performance differences decreased with practice. This suggests that the participants might have conceptualized the two tasks as belonging together. The current experiments confirm this assumption by additionally showing that the stimulus of one task seems to re-activate the responses of both tasks.

The current findings are also in line with the conclusions that can be drawn from experiments investigating sequence learning under dual-task conditions. More indirectly, this line of research also suggests that task integration plays a role in dual-tasking. Evidence stems from the observation that sequence learning is hampered whenever the stimuli of the second task are randomly presented and both tasks stimuli appear in close temporal proximity (Hsiao & Reber, 2001; Roettger et al., 2019; Schmidtke & Heuer, 1997; Schumacher & Schwarb, 2009). The most direct evidence was provided by Röttger et al. (2021) who showed that implicit learning was robustly observable if the two tasks within a trial could be integrated, whereas it was reduced when impeding such an integration. Both these lines of empirical findings provide converging support for our assumption that the elements of the two tasks are integrated into one conjoint representation.

In summary, we provided evidence that the two tasks in dual-tasking are likely not represented separately as two entirely distinct task sets but as one integrated representation of both tasks. This is in line with the emerging shift in the focus of dual-tasking research away from the parallel versus serial processing debate to the question of what is actually represented as a task in a dual-tasking situation (Künzell et al., 2018; Schumacher & Hazeltine, 2016).

## Data Availability

The merged raw data are available at https://osf.io/ze27t/

## Footnotes

1 Moeller and Frings (2019) found response repetition effects with rather similar types of matching tasks.

2 We did not exclude trials for which an error occurred in Trial n-1. We analyzed the data in all three experiments by additionally excluding all trials with Trial n-1errors. This did not change the pattern of the results.

3 As we found large performance differences between FRs and PRs in previous studies, we expected to find also a large effect in the present study. However, as we changed the experimental design, we used the standard large effect size of *ηp*^2^ = 0.14 (Cohen, 1998) to calculate the sample size needed to reliably detect this effect at 90% power (alpha = 0.05) in the present study.

## References

[CR1] Cohen J (1998). Statistical power analysis for the behavioral sciences.

[CR2] Dreisbach G (2012). Mechanisms of cognitive control: The functional role of task rules. Current Directions in Psychological Science.

[CR3] Dreisbach G, Haider H (2008). That’s what task sets are for: Shielding against irrelevant information. Psychological Research Psychologische Forschung.

[CR6] Freedberg M, Wagschal TT, Hazeltine E (2014). Incidental learning and task boundaries. Journal of Experimental Psychology: Learning, Memory, and Cognition.

[CR7] Frings C, Koch I, Rothermund K, Dignath D, Giesen C, Hommel B, Möller M (2020). Merkmalsintegration und abruf als wichtige prozesse der handlungssteuerung–eine paradigmen-übergreifende perspektive. Psychologische Rundschau.

[CR8] Gade M, Souza AS, Druey MD, Oberauer K (2017). Analogous selection processes in declarative and procedural working memory: N-2 list-repetition and task-repetition costs. Memory & Cognition.

[CR9] Geyer T, Müller HJ, Krummenacher J (2006). Cross-trial priming in visual search for singleton conjunction targets: Role of repeated target and distractor features. Perception & Psychophysics.

[CR10] Greenwald AG (1970). Sensory feedback mechanisms in performance control: With special reference to the ideo-motor mechanism. Psychological Review.

[CR101] Henson RN, Eckstein D, Waszak F, Frings C, Horner AJ (2014). Stimulus–response bindings in priming. Trends in Cognitive Cciences.

[CR11] Hillstrom AP (2000). Repetition effects in visual search. Perception & Psychophysics.

[CR12] Hirsch P, Nolden S, Koch I (2017). Higher-order cognitive control in dual tasks: Evidence from task-pair switching. Journal of Experimental Psychology: Human Perception and Performance.

[CR13] Hirsch P, Nolden S, Philipp AM, Koch I (2018). Hierarchical task organization in dual tasks: Evidence for higher level task representations. Psychological Research Psychologische Forschung.

[CR14] Hirsch P, Roesch C, Koch I (2021). Evidence for a multicomponent hierarchical representation of dual tasks. Memory & Cognition.

[CR15] Hommel B (1998). Event files: Evidence for automatic integration of stimulus-response episodes. Visualcognition.

[CR16] Hommel B, Colzato LS (2009). When an object is more than a binding of its features: Evidence for two mechanisms of visual feature integration. Visual Cognition.

[CR17] Hommel B, Frings C (2020). The disintegration of event files over time: Decay or interference?. Psychonomic Bulletin & Review.

[CR18] Hommel B, Müsseler J (2006). Action-feature integration blinds to feature-overlapping perceptual events: Evidence from manual and vocal actions. Quarterly Journal of Experimental Psychology.

[CR19] Hsiao AT, Reber AS (2001). The dual-task SRT procedure: Fine-tuning the timing. Psychonomic Bulletin & Review.

[CR20] Huestegge L, Hoffmann MA, Strobach T (2021). Task-order representations in dual tasks: Separate or integrated with component task sets?. Quarterly Journal of Experimental Psychology.

[CR21] Janczyk M, Kunde W (2020). Dual tasking from a goal perspective. Psychological Review.

[CR106] JASP Team. (2020).* JASP* (Version 0.12.2). https://jasp-stats.org/.

[CR105] Jeffreys H (1961). Theory of probability.

[CR22] Kahneman D (1973). Attention and effort.

[CR103] Koch I, Frings C, Schuch S (2017). Explaining response-repetition effects in task switching: Evidence from switching cue modality suggests episodic binding and response inhibition. Psychological Research.

[CR24] Koch I, Keller P, Prinz W (2004). The ideomotor approach to action control: Implications for skilled performance. International Journal of Sport and Exercise Psychology.

[CR25] Koch I, Poljac E, Müller H, Kiesel A (2018). Cognitive structure, flexibility, and plasticity in human multitasking—an integrative review of dual-task and task-switching research. Psychological Bulletin.

[CR26] Kübler, S. (2021). Investigating task-order coordination in dual-task situation. PhD-Thesis. Berlin, 2021.

[CR27] Kübler S, Reimer CB, Strobach T, Schubert T (2018). The impact of free-order and sequential-order instructions on task-order regulation in dual tasks. Psychological Research Psychologische Forschung.

[CR28] Kübler S, Strobach T, Schubert T (2021). The role of working memory for task-order coordination in dual-task situations. Psychological Research Psychologische Forschung.

[CR29] Kunde W, Koch I, Hoffmann J (2004). Anticipated action effects affect the selection, initiation, and execution of actions. Quarterly Journal of Experimental Psychology: Human Experimental Psychology.

[CR30] Künzell S, Broeker L, Dignath D, Ewolds H, Raab M, Thomaschke R (2018). What is a task? An Ideomotor Perspective. Psychological Research Psychologische Forschung.

[CR31] Lamy D, Zivony A, Yashar A (2011). The role of search difficulty in intertrial feature priming. Vision Research.

[CR33] Logan GD, Gordon RD (2001). Executive control of visual attention in dual-task situations. Psychological Review.

[CR34] Luria R, Meiran N (2003). Online order control in the psychological refractory period paradigm. Journal of Experimental Psychology: Human Perception and Performance.

[CR35] Luria R, Meiran N (2006). Dual route for subtask order control: Evidence from the psychological refractory paradigm. Quarterly Journal of Experimental Psychology.

[CR37] Meyer DE, Kieras DE (1997). A computational theory of human multiple task performance: The EPIC information-processing architecture and strategic response deferment model. Psychological Review.

[CR39] Moeller B, Frings C (2019). From simple to complex actions: Response–response bindings as a new approach to action sequences. Journal of Experimental Psychology: General.

[CR40] Navon D, Miller J (2002). Queuing or sharing? A critical evaluation of the single-bottleneck notion. Cognitive Psychology.

[CR41] Nissen MJ, Bullemer P (1987). Attentional requirements of learning: Evidence from performance measures. Cognitive Psychology.

[CR42] Palan S, Schitter C (2018). Prolific. Ac—a subject pool for online experiments. Journal of Behavioral and Experimental Finance.

[CR43] Pashler H (1994). Dual-task interference in simple tasks: Data and theory. Psychological Bulletin.

[CR44] Peirce J, Gray JR, Simpson S, MacAskill M, Höchenberger R, Sogo H, Lindeløv JK (2019). PsychoPy2: Experiments in behavior made easy. Behavior Research Methods.

[CR45] Pelzer L, Naefgen C, Gaschler R, Haider H (2021). Learning of across- and within-task contingencies modulates partial-repetition costs in dual-tasking. Psychological Research Psychologische Forschung.

[CR46] Prinz W, Heuer H, Sanders AF (1987). Ideo-motor action. Perspectives on perception and action.

[CR47] Rah SKY, Reber AS, Hsiao AT (2000). Another wrinkle on the dual-task SRT experiment: It’s probably not dual task. Psychonomic Bulletin & Review.

[CR48] Reber, A. (1993). *Implicit learning and tacit knowledge: An essay on the cognitive unconscious*. Oxford University Press. 10.1093/acprof:oso/9780195106589.001.0001.

[CR49] Röttger E, Haider H, Zhao F, Gaschler R (2019). Implicit sequence learning despite multitasking: The role of across-task predictability. Psychological Research Psychologische Forschung.

[CR50] Röttger E, Zhao F, Gaschler R, Haider H (2021). Why does dual-tasking hamper implicit sequence learning?. Journal of Cognition.

[CR104] Rouder JN, Speckman PL, Sun D, Morey RD, Iverson G (2009). Bayesian t tests for accepting and rejecting the null hypothesis. Psychonomic Bulletin & Review.

[CR51] Schmidtke V, Heuer H (1997). Task integration as a factor in secondary-task effects on sequence learning. Psychological Research Psychologische Forschung.

[CR102] Schuch S, Koch I (2004). The costs of changing the representation of action: response repetition and response-response compatibility in dual tasks. Journal of Experimental Psychology: Human Perception and Performance.

[CR52] Schumacher EH, Hazeltine E (2016). Hierarchical task representation: task files and response selection. Current Directions in Psychological Science.

[CR53] Schumacher EH, Schwarb H (2009). Parallel response selection disrupts sequence learning under dual-task conditions. Journal of Experimental Psychology: General.

[CR54] Sigman M, Dehaene S (2006). Dynamics of the central bottleneck: Dual-task and task uncertainty. PLoS Biology.

[CR55] Stelzel C, Kraft A, Brandt SA, Schubert T (2008). Dissociable neural effects of task order control and task set maintenance during dual-task processing. Journal of Cognitive Neuroscience.

[CR56] Strobach T, Hendrich E, Kübler S, Müller H, Schubert T (2018). Processing order in dual-task situations: The “first-come, first-served” principle and the impact of task order instructions. Attention, Perception, & Psychophysics.

[CR57] Strobach T, Kübler S, Schubert T (2021). Endogenous control of task-order preparation in variable dual tasks. Psychological Research Psychologische Forschung.

[CR59] Telford CW (1931). The refractory phase of voluntary and associative responses. Journal of Experimental Psychology.

[CR60] Tombu M, Jolicœur P (2003). A central capacity sharing model of dual-task performance. Journal of Experimental Psychology: Human Perception and Performance.

[CR61] Welford AT (1952). The “psychological refractory period” and the timing of high-speed performance—a review and a theory. British Journal of Psychology.

[CR62] Zehetleitner M, Rangelov D, Müller HJ (2012). Partial repetition costs persist in nonsearch compound tasks: Evidence for multiple-weighting-systems hypothesis. Attention, Perception, & Psychophysics.

[CR63] Zhao F, Gaschler R, Nöhring DO, Röttger E, Haider H (2020). Sequential modulation of across-task congruency in the serial reaction time task. Acta Psychologica.

